# Genetic and Pathogenic Overlaps Between Autism Spectrum Disorder and Alzheimer’s Disease: Evolutionary Features and Opportunities for Drug Repurposing

**DOI:** 10.3390/ijms262010066

**Published:** 2025-10-16

**Authors:** Ekaterina A. Trifonova, Anna A. Pashchenko, Roman A. Ivanov, Alex V. Kochetov, Sergey A. Lashin

**Affiliations:** 1 Federal Research Center Institute of Cytology and Genetics, Siberian Branch of the Russian Academy of Sciences, 630090 Novosibirsk, Russia; ivanovromanart@bionet.nsc.ru (R.A.I.); ak@bionet.nsc.ru (A.V.K.); lashin@bionet.nsc.ru (S.A.L.); 2 Natural Science Faculty, Novosibirsk National Research State University, 630090 Novosibirsk, Russia

**Keywords:** genetics, bioinformatics, autism spectrum disorder (ASD), Alzheimer’s disease (AD), mTOR signaling pathway, Phylostratigraphic Age Index (PAI), Divergence Index (DI), drug repurposing

## Abstract

Autism spectrum disorder (ASD) and Alzheimer’s disease (AD) are neurodevelopmental and neurodegenerative disorders, respectively. While exome sequencing is routinely employed during the early stages of ASD diagnosis, it rarely influences therapeutic strategies. To address this gap, we have reconstructed and analyzed the gene networks linking autism spectrum disorders, Alzheimer’s disease, and mTOR signaling. In addition, we have performed a phylostratigraphic analysis that reveals similarities and differences in the evolution of both ASD and Alzheimer’s disease predisposition genes. We have shown that almost half of the genes predisposing to autism and two-fifths of the genes predisposing to Alzheimer’s disease are directly related to the mTOR signaling pathway. Analysis of Phylostratigraphic Age Index (PAI) value distributions revealed a significant enrichment of evolutionarily ancient genes in both ASD- and AD-related gene sets. When studying the distribution of ASD predisposition genes by Divergence Index (DI) values, a significant enrichment with genes having extremely low DI = 0 has been found. Such low DI values indicate that most likely these genes are under stabilizing selection. Using the ANDVisio tool, both pharmacological and natural mTOR regulators with potential for ASD treatment were selected, such as propofol, dexamethasone, celecoxib, statins, berberine, resveratrol, quercetin, myricetin, mio-inositol, and several amino acids.

## 1. Introduction

Autism spectrum disorder (ASD) and Alzheimer’s disease (AD) are neurological disorders affecting young and elderly people, respectively. According to data from the Centers for Disease Control and Prevention (CDC), ASD affects about 1 in 31 (3.2%) children aged 8 years in the USA [1]. The diagnostic criteria of the disorder: 1. Impaired communication, including verbal communication, and socialization, 2. Stereotypical interests, speech, and behavior, often accompanied by hypersensitivity. Alzheimer’s disease (AD) is characterized by cognitive impairment and memory loss in the elderly. An estimated 5.8 million U.S. citizens aged 65 and older are struggling with the disease [2,3]. Both ASD and AD are often accompanied by reduced cognitive functions, language impairment, depression, irritability, and aggression [4].

Immune system abnormalities and autoimmunity have been regularly reported in both ASD and AD patients. The hypothesis that a certain part of the autistic spectrum may be an autoimmune disorder has been discussed repeatedly [5,6,7]. Recently, this idea has been reinforced by the discovery of pronounced perivascular lymphocytic infiltrates and related astrocytic blebs within Virchow–Robin spaces and subarachnoid cerebrospinal fluid (CSF) in ~65% of ASD patients. The revealed pathology indicates that dysregulated cellular immunity damages astrocytes in foci along the CSF–brain barrier in ASD [6,8].

At the same time, Alzheimer’s disease may also be considered as an autoimmune disease of innate immunity [9]. In response to various initiating stimuli (for example, infection, trauma, and ischemia), Aß is released as an early response immunopeptide, triggering a cascade of innate immunity in which Aß exhibits both immunomodulatory and antimicrobial properties (regardless of whether bacteria are present or not), which leads to an attack of its own neurons. The similar electronegative topology of the membrane surfaces of neurons and bacteria makes them equally susceptible to membrane penetration by antimicrobial peptides such as Aß. After the attack, the products of neuronal necrotic decay diffuse into neighboring neurons, causing further release of Aß, which leads to a chronic self-reproducing autoimmune cycle [10].

Mechanism-based treatments for ASD often target neuroinflammation, immune dysfunction, mitochondrial impairment, and the deregulated mTOR signaling pathway [11]. In fact, gene mutations and/or dysregulation of the mTOR signaling pathway may be responsible for both immune disorders and mitochondrial dysfunction. It has been shown [12,13] that activation of the mTOR signaling pathway is a positive regulator of CD4+ effector T cell differentiation and a negative regulator of Treg (regulatory T cell) differentiation, and that mTOR hyperactivation is associated with the pathogenesis of a number of autoimmune diseases.

At the same time, mitochondrial dysfunction is increasingly being considered as one of the causes of the neurodevelopmental disorders, including ASD [14]. In animal models of tuberous sclerosis (Tsc2+/−), one of the variants of syndromic autism, it has been shown that the regulation of the mitochondrial life cycle through autophagy, or mitophagy, is critically impaired both in the axon and throughout the body. A treatment of Tsc2-deficient neurons and model animals with the mTOR inhibitor rapamycin restored the mitochondrial phenotype, including mass, transport, and mitophagy [15]. Thus, reducing the activity of the mTOR pathway has become the primary pharmacological strategy for activating autophagy [16].

The discovery that the powerful mTOR inhibitor rapamycin increases the lifespan of mice and restores/slows down aging phenotypes has led to the assumption that rapamycin has “rejuvenating” properties. The main question is whether the manipulation of anti-aging rapamycin’s properties can affect the onset and/or progression of Alzheimer’s disease [17].

Since rapamycin is known to induce autophagy—a primary cellular mechanism for degrading protein aggregates—its potential to reduce amyloid plaques and neurofibrillary tangles was investigated in AD mouse models. It was shown that rapamycin-induced autophagy plays an important role in reducing the accumulation of plaques and tangles in the brains of AD model mice and possibly in improving memory [17,18].

To assess the genetic and pathogenic similarity between ASD and AD, we have reconstructed and analyzed the gene networks linking autism spectrum disorders, Alzheimer’s disease, autoimmunity, and the mTOR signaling pathway. Additionally, we have performed a phylostratigraphic analysis, which estimates the evolutionary origin of genes by examining phylogenetic trees of organisms with orthologous genes. Such an analysis may reveal similarities and differences in the evolution of both ASD and Alzheimer’s disease predisposition genes, as well as the genes of the mTOR signaling pathway. In addition to investigating the time of gene origin, we have also evaluated the genetic variability and the type of natural selection acting upon these gene sets. Based on the analysis, we have tried to explore approaches for applying the therapeutic solutions that have been obtained in a significant number for AD to ASD.

## 2. Results

### 2.1. SFARI Gene Database and AD Genes Comparative Gene-Set and Pathway Analysis

**Figure 1:**
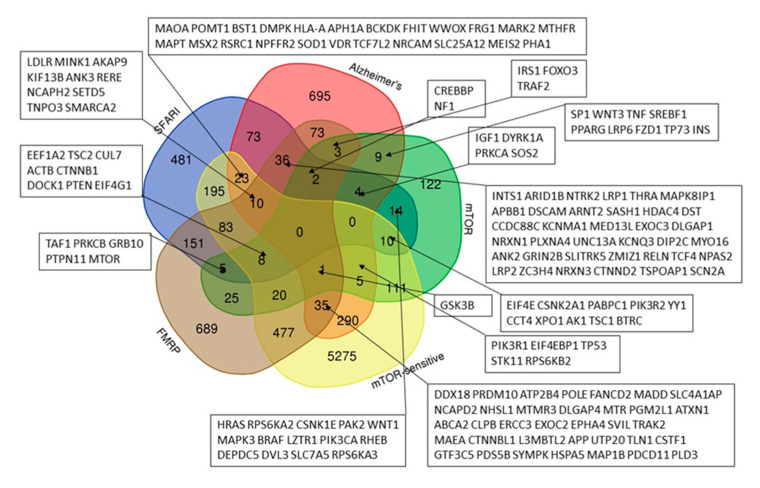
A Venn diagram representing the intersections of five sets of genes: genes from the SFARI Gene database, a combined set of Alzheimer’s disease genes, genes of the mTOR signaling pathway, mTOR-sensitive genes and FMRP target genes.

Our analysis (Figure 1) has shown that the sets of genes predisposing to ASD (from the SFARI database) and to Alzheimer’s disease have 148 common genes, out of which 75 are directly related to mTOR signaling pathway. In total, 49.4% (541 out of 1095) of the genes from the SFARI database and 39.0% (491 out of 1259) of the AD genes are associated with mTOR, which allows us to assume these diseases as mTORopathies. Specifically, we have shown that both ASD (from the SFARI database) and AD predisposing genes can be attributed to one of three groups: 1. mTOR signaling pathway genes, 2. mTOR-modulated genes, 3. FMRP target genes.

The FMRP, a negative regulator of translation initiation, is one of the key components of the local translation system. Since FMRP is the target of the S6K1 kinase [19], which is an element of the mTOR pathway, the translation regulated by FMRP is also always dependent on mTOR. The complete lists of all analyzed gene sets are given in Supplementary Table S1.

### 2.2. Analysis of Evolutionary Characteristics of Gene Sets

#### 2.2.1. Phylostratigraphic Age of Genes (PAI-Based Analysis)

**Figure 2:**
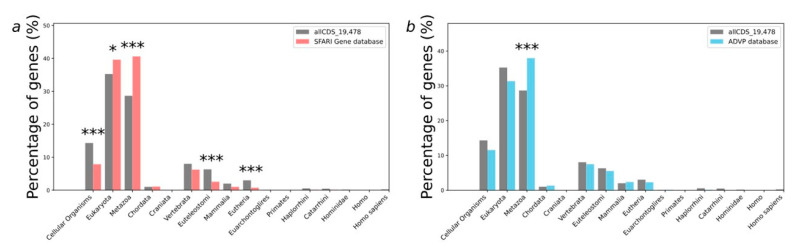
Distributions by PAI values obtained for the sets of human genes implicated to autism susceptibility and Alzheimer’s disease predisposition. (**a**) All human protein-coding genes as a control group compared to genes implicated to autism susceptibility from SFARI database; (**b**) all human protein-coding genes as a control group compared to Alzheimer’s disease predisposition genes. Columns marked with asterisks indicate statistically significant differences between gene samples: * *p*-value < 0.05, *** *p*-value < 0.001. Statistical testing was performed using the chi-square test with Benjamini–Hochberg adjustment for multiple analysis.

We have analyzed the distribution of PAI values for all human protein-coding genes (the allCDS_19478 set) as a control group compared to genes implicated in autism susceptibility from the SFARI database and Alzheimer’s disease predisposition genes (Figure 2a,b). In addition, we have compared neurodevelopmental disorder predisposition genes as a control group to autism predisposition genes of the largest cohort of autistic patients [20] (Supplementary Figure S1).

We have found that the PAI values are unevenly distributed (Figure 2a,b). 28.6% of all control allCDS_19478 gene sets had a PAI equal to 3 (Metazoa, multicellular, eukaryotic organisms in the biological kingdom Animalia), and the proportions of genes that have had PAI values equal to 1 (cellular organisms), 7 (the stage of euteleostomi divergence), and 9 (the stage of eutheria, also called Pan-Placentalia, divergence) were 14.3%, 6.3%, and 3.0%, respectively. When we examined the distribution of PAI values for a set of autism predisposition genes from the SFARI Gene database, we found that 40.6% of the SFARI genes had a PAI equal to 3 (Metazoa), 7.9% equal to 1 (cellular organisms), 2.6% equal to 7 (euteleostomi), and 0.7% equal to 9 (eutherian). All these numbers are significantly different (*p* < 0.001) from the expected numbers, calculated based on the distribution obtained for the allCDS_19478 gene set (Figure 2a). In contrast, PAI values distribution for Alzheimer’s disease predisposition genes is greatly different from the control allCDS_19478 gene set only for a PAI equal to 3 (Metazoa) for *p*-value < 0.001. Thus, 37.9% of Alzheimer’s disease predisposition genes from the ADVP database have had a PAI equal to 3 (Figure 2b).

Next, we have compared the distribution of PAI values for 185 autism predisposition genes and 664 autism and developmental delay predisposition genes (ASD/DD) from the largest cohort of autistic patients [20]. Surprisingly, we have found no significant differences between these sets of genes in the distributions of PAI values (Supplementary Figure S1).

#### 2.2.2. Evolutionary Variability of Genes (DI-Based Analysis)

**Figure 3:**
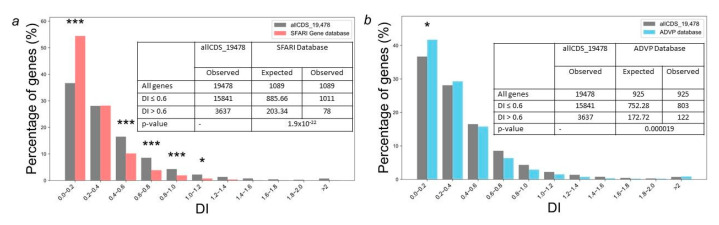
Distributions of genes from the sets of human genes implicated in autism susceptibility and Alzheimer’s disease predisposition according to the DI. (**a**): All human protein-coding genes as a control group compared to genes implicated in autism susceptibility from the SFARI database; (**b**): All human protein-coding genes as a control group compared to Alzheimer’s disease predisposition genes. Columns marked with asterisks indicate statistically significant differences between gene samples: * *p*-value < 0.05, *** *p*-value < 0.001. Statistical testing was performed using the chi-square test with Benjamini–Hochberg adjustment for multiple analysis.

The analysis of gene distribution from the SFARI Gene database based on DI values (Figure 3a) showed that 54.5% of genes had DI < 0.2, most genes (~98.6%) had DI < 1, and only 13 out of 1095 genes had DI > 1, indicating that most of the genes are under stabilizing selection.

A comparison of gene distribution from the SFARI Gene database by DI values with the distribution obtained for all genes encoding human proteins (allCDS_19478 set) has shown that SFARI Gene database genes are characterized by an increased content of genes with extremely low DI values (Figure 3a). A total of 54.5% from SFARI Gene database have had DI < 0.2, which is significantly (*p* < 0.001) higher than the expected number (36.7%) calculated using the distribution obtained for all human protein-coding genes (Figure 3a). This trend is even more significant for sets of autism and neurodevelopmental disorder predisposition genes obtained from the largest datasets [20]. In total 66.9% of ASD and 70.2% of ASD/DD predisposition genes had DI < 0.2, and no one gene from these sets had DI > 1 (Supplementary Figure S1). By contrast, the DI distribution for Alzheimer’s disease predisposition genes from the ADVP database [21] was only slightly different from all genes encoding human proteins in the allCDS_19478 set (Figure 3b).

We have also compared average values of PAI and DI indices for the most relevant sets of genes listed in the Materials and Methods section (Table 1). The results have shown significant differences in the average values of PAI and DI indices for all genes associated with ASD and ASD/DD compared to all protein-coding human genes. These differences have been more pronounced than those observed for Alzheimer’s disease predisposition genes. The complete lists of evolutionary characteristics (both PAI and DI indices) for all analyzed gene sets are given in Supplementary Table S2.

### 2.3. Comparative Network Analysis of Genes Predisposing to Autism and Alzheimer’s Disease with Genes of Autoimmune Diseases

**Figure 4:**
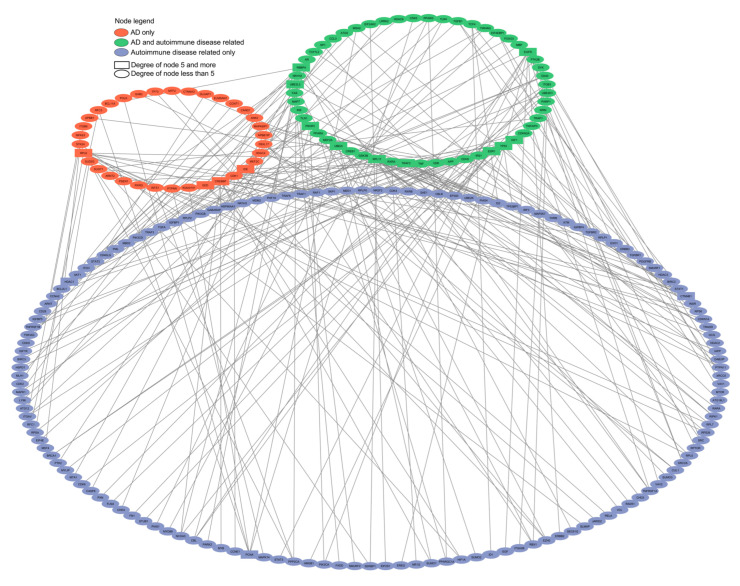
A gene network representing genes predisposed to Alzheimer’s disease and genes of autoimmune disorders. The left legend shows the color and degree of the nodes.

We have reconstructed a gene network based on sets of genes predisposing to Alzheimer’s disease (gene sets 4 and 5) and genes associated with autoimmune diseases (gene set 9). An initial set of 4993 genes (808 genes of predisposition to AD, 3734 genes of autoimmune diseases, and 451 genes belonging to both sets) has been analyzed using stringApp for Cytoscape 3.10.4. In Cytoscape, all edges of the network have been removed, except for those indicating experimentally established interactions with a confidence level of more than 0.9. As a result, a gene network consisting of 230 genes and 229 connections between them has been obtained (Figure 4). Each gene is color-coded according to its originating set.

The products of this network’s most connected genes include ribosomal proteins (RPL6, RPL17) [25], transcription factors and coregulatory proteins (EED, TP53, ESR1, CREBBP, RBBP4, HDAC1) [26,27,28], ubiquitinating proteins (UBE2L3, UBE2I), signaling pathway proteins (TRAF2, CTNNB1, PIK3R1), as well as insulin-destroying enzyme (IDE), insulin-like growth factor (IGF1), epidermal growth factor receptor (EGFR), and a protein involved in DNA replication and repair (PCNA).

**Figure 5:**
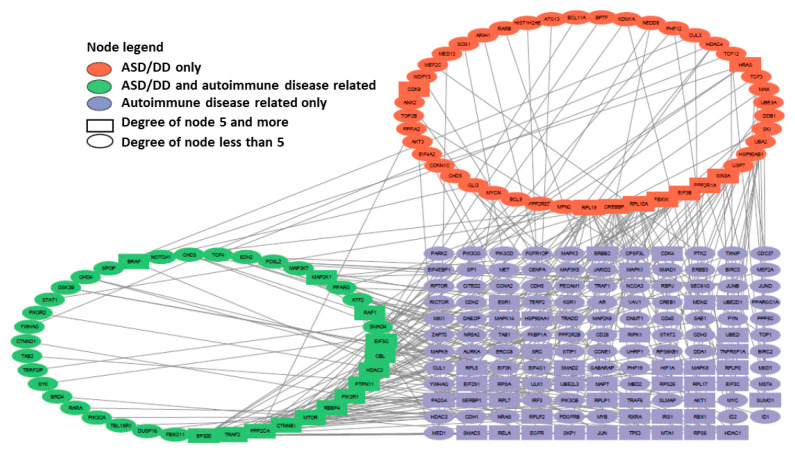
A gene network representing genes predisposing to autism and developmental delay (ASD/DD) and genes of autoimmune disorders. The left legend shows the color and degree of the nodes.

We have also reconstructed a gene network for sets of genes predisposing to autism and developmental delay (ASD/DD) (gene set 3) and genes associated with autoimmune disorders (gene set 9). The initial set of 4684 genes (499 genes for predisposition to ASD/DD, 4020 autoimmune disease genes, and 165 genes belonging to both sets) has been analyzed using stringApp for Cytoscape. In Cytoscape, all edges of the network were removed, except for those indicating experimentally established interactions with a confidence level of more than 0.9. As a result, a gene network consisting of 215 genes and 233 connections between them has been obtained (Figure 5). Genes are color-coded based on their source dataset.

The products of the most connected genes of this network include coregulatory proteins (CREBBP, RBBP4, HDAC1, HDAC2, and EP300) [28,29,30], signaling pathway proteins (TRAF2, CTNNB1, and PIK3R1), proteins involved in ubiquitination (FBXW7 and CBL), phosphatases (PPP2CA and PTPN11), ribosomal protein (RPL10A), translation initiation factor (EIF3G), as well as directly the mTOR protein (MTOR). Of all the most connected genes in this gene network, only the HDAC1 gene does not belong to the set of genes predisposing to ASD/DD.

**Figure 6:**
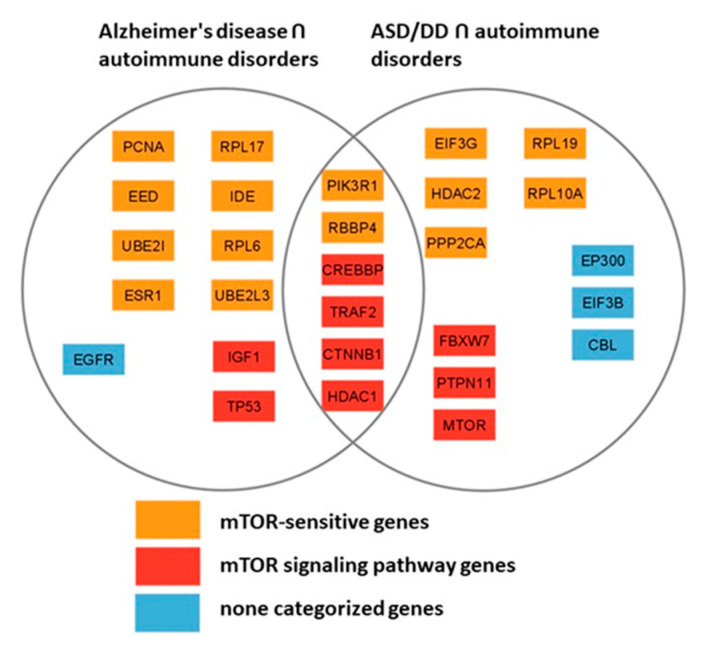
Comparison of the most connected genes from the gene networks representing the interactions of genes predisposing to Alzheimer’s disease and genes of autoimmune disorders (Figure 4) and genes associated with autism and developmental delay (ASD/DD) and genes of autoimmune disorders (Figure 5).

The two reconstructed networks are comparable in size and connectivity, each containing a substantial number of genes with a node degree of 5 or higher. Among the most connected products of the genes of these networks, 6 are common (PIK3R1, RBBP4, CREBBP, TRAF2, CTNNB1, and HDAC1), 15 are mTOR-sensitive, 9 relate directly to the mTOR signaling pathway. Only 1 gene product from Figure 4 network (EGFR) and 3 gene products from Figure 5’s network (EP300, EIF3B, and CBL) do not belong to either of the two groups, which indicates the key role of the mTOR signaling pathway in the pathogenesis of autoimmune diseases, AD, and neurodevelopmental disorders, including ASD (Figure 6).

### 2.4. The Associative Network Analysis of the Main Elements of the mTOR Pathway and Substances Regulating Their Activity Using for ASD and AD Treatment

**Figure 7:**
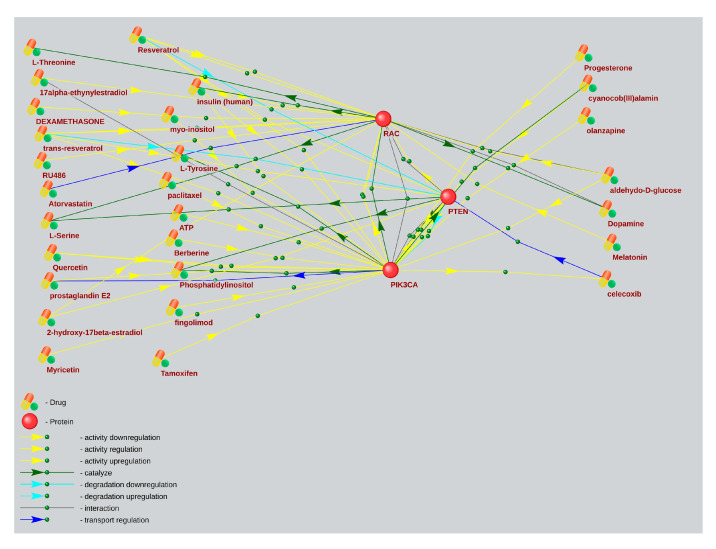
An associative gene network representing the interactions of the main elements of the PI3K/Akt pathway and substances regulating their activity. On the left are drug-type objects associated with Alzheimer’s disease but not associated with ASD; on the right are drug-type objects associated with ASD.

**Figure 8:**
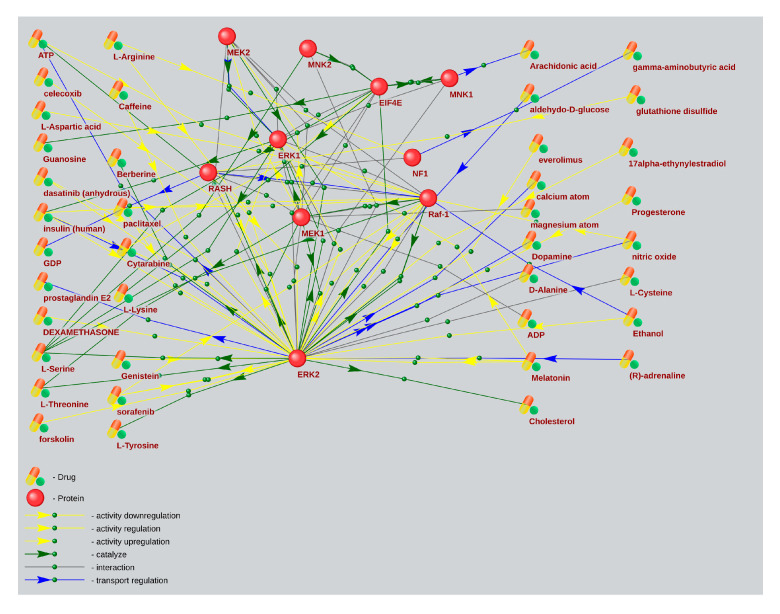
An associative gene network representing the interactions of the main elements of the Ras pathway and substances regulating their activity. On the left are drug-type objects associated with Alzheimer’s disease but not associated with ASD; on the right are drug-type objects associated with ASD.

**Figure 9:**
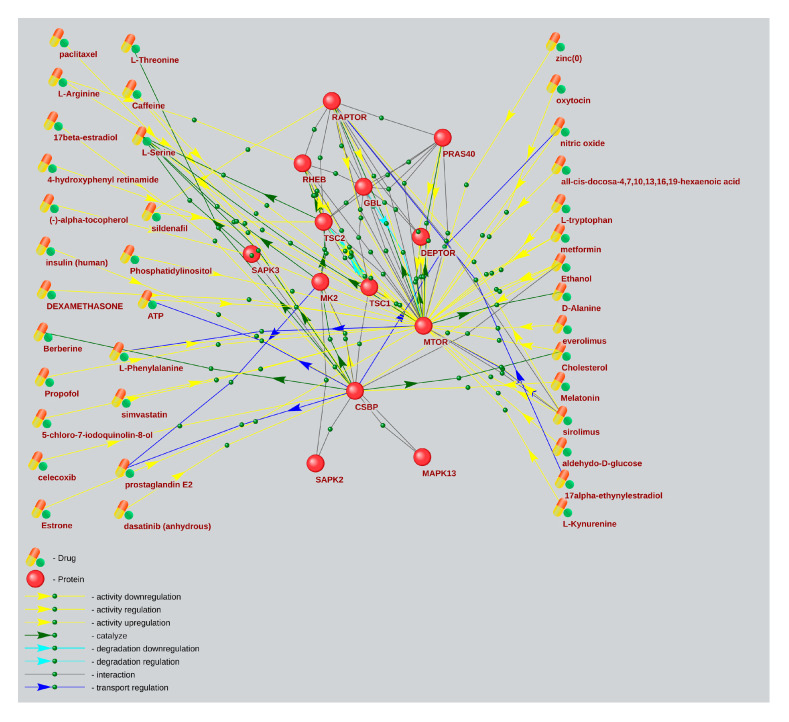
An associative gene network representing the interactions of the main elements of the p38 MAPK pathway and substances regulating their activity. On the left are drug-type objects associated with Alzheimer’s disease but not associated with ASD; on the right are drug-type objects associated with ASD.

Based on a large percentage and high degree of connectivity of mTOR-related ASD and AD genes (Figure 1 and Figure 6), we have searched for mTOR inhibitors used in AD therapy and with potential for ASD therapy by means of the ANDVisio tool. For this purpose, the ANDVisio tool has been used to construct associative networks based on three major fragments of the mTOR pathway: PI3K/Akt (Figure 7), Ras (Figure 8), and p38 MAPK (Figure 9). Drug-type objects associated with ASD and AD have been added to each graph, and those that had an association with AD but no association with ASD have been identified.

What is notable drug-type objects associated with Alzheimer’s disease significantly have outnumbered drug-type objects associated with ASD in all the associative networks we have reconstructed.

## 3. Discussion

Initially, pharmacological mTOR signaling inhibitors have been used after transplantation and cancer treatment. However, it has been recently shown that aberrant protein aggregation, such as deposition of beta-amyloid (Aß) and tau filaments, as well as cognitive impairment, are chased away by the inhibition of mTOR [31]. As early as 2008, it was first shown that adult Tsc2+/− mice, one of the most common syndromic autism animal models, practically overcame both behavioral deficits and learning problems after a short course of rapamycin [32].

Keyword search for “Alzheimer’s disease” + “treatment” in the PubMed database (https://pubmed.ncbi.nlm.nih.gov) returned 86,391 results, while the same search for “autism” + “treatment” returned only 26,778 results. Moreover, since autism is exclusively diagnosed according to behavioral criteria that overlook clinical and genomic heterogeneity, some publications contain information concerning failed clinical trials [33].

The analyses presented here suggest that dysregulation of the mTOR pathway could serve as a promising criterion for subtyping ASD in order to propose the optimal treatment option. Thus, we have shown that more than half of the genes common to autism and Alzheimer’s disease are associated with mTOR signaling, and that almost half of the genes predisposing to autism and two-fifths of the genes predisposing to Alzheimer’s disease are directly related to the same signaling pathway (Figure 1).

The gene networks linking Alzheimer’s disease and autism with autoimmune disorders have allowed us to identify the most connected genes from these sets; more than half of them are mTOR-sensitive, a third belong to the mTOR signaling pathway itself, and only 4 genes are not directly related to the signaling pathway. Collectively, these findings confirm the pivotal role of the mTOR signaling pathway in the pathogenesis of not only autoimmune pathologies, but also AD and ASD (Figure 6).

The examination of the distributions of AD and ASD predisposition genes by PAI values has revealed that: (1) both Alzheimer’s disease (from the ADVP database) and autism (from SFARI Gene database) genes have a significantly increased content of genes with the same phylostratigraphic age (PAI = 3, Metazoa, multicellular, eukaryotic organisms in the biological kingdom Animalia) than all protein coding genes; (2) ASD predisposition genes (from SFARI Gene database) are much more different from control allCDS_19478 gene set than Alzheimer’s disease (from the ADVP database) in the distribution of PAI values; (3) ASD predisposition genes (from SFARI Gene database) have had a significantly enriched content of evolutionarily ancient genes; (4) average values of PAI for Alzheimer’s disease genes (from the ADVP database), ASD genes (from SFARI Gene database) and mTOR signaling pathway are 3.18, 2.86, and 2.29, respectively (Table 1). Such low PAI values are typical for specific types of cancers; as for neurodegenerative diseases, they are close to the value for Parkinson’s disease [34]. The fact that mTOR signaling pathways have had even greater enrichment of evolutionarily ancient genes with average values of PAI 2.29 indirectly supports the hypothesis of the link between mTOR and autism, and to a lesser extent Alzheimer’s disease.

When examining the distribution of ASD predisposition genes from gene sets 1 and 2 by DI values, a significant enrichment of this group with genes subjected to stabilizing selection has been revealed. An extremely low DI value (=0) has been found for 66 genes, or 6% of the SFARI Gene database (gene set 1), and 14 genes (ACVR2A, AP2S1, CAMK2A, CTCF, CUL3, HNRNPD, MARK2, NACC1, OBSCN, SATB2, SLC6A1, SPAST, TBR1, and TLE3), or 8% of gene set 2. Moreover, not a single gene among the ASD predisposition genes from gene set 2 had a DI > 1 (Supplementary Table S2), indicating that these gene sets have been subject to stabilizing selection. At the same time, fifteen ASD predisposition genes from the SFARI gene database (gene set 1) had high DI values (DI > 1): DYDC2, CX3CR1, OR1C1, CHM, DDX53, LAS1L, RAD21L1, SPP2, CYLC2, SLC25A39, DYDC1, MACROD2, KCNK7, GSTM1, and CD99L2. Only one gene (DYDC2) among the genes had a DI as high as 3, indicating that this gene has probably been subjected to strong driving selection. DYDC2 and DYDC1 (DI > 1) encode proteins that contain a DPY30 domain, which is necessary for the methylation of histone H3 [35]. The proteins are involved in spermatogenesis and acrosome formation. Northern blot analysis detected DYDC1 and DYDC2 expression in the testis and brain only; yet, the function of the genes in the brain is unknown [36].

We have also found that the distribution of genes from the ADVP database (gene set 5) by DI values is very slightly different from the control allCDS_19478 gene set (Figure 3). An extremely low DI value (=0) has been found for 26 genes, or 3% of Alzheimer’s disease genes. Notably, the most connected genes in the gene network representing genes predisposing to Alzheimer’s disease and genes associated with autoimmune disorders contain a significantly increased number of genes with low DI values. Nine out of the seventeen most connected genes (RPL6, RPL17, EED, RBBP4, HDAC1, UBE2L3, UBE2I, CTNNB1, PCNA) have had the lowest DI values (DI < 0.05) (Figure 4 and Supplementary Table S2).

We have also examined the distribution of the most connected genes from two gene networks (Figure 4 and Figure 5, generalized Figure 6) by DI values and have found that sixteen out of the twenty-eight genes have had an extremely low DI value (<0.05). The data we have obtained suggest that perhaps the most connected genes in any gene network tend to have a very low DI value and therefore undergo stabilizing selection. The pressures of stabilizing selection are usually associated with highly conservative traits, such as biochemical metabolic pathways, which are similar in most taxa. One of these pathways is definitely mTOR. The significant prevalence of genes with an extremely low DI = 0 among ASD-associated genes once again suggests a metabolic basis for the disorder.

The characteristic features of ASD and Alzheimer’s disease predisposition genes distribution according to the PAI and DI indices have revealed in this study provide a starting point for further analyses of the evolutionary characteristics of the entire gene networks associated with both neurodevelopmental and neurodegenerative diseases.

The ANDVisio is a bioinformatic tool designed to reconstruct, visualize, and analyse associative gene networks in the previously developed Associative Network Discovery System (ANDSystem) software (https://www-bionet.sscc.ru/andvisio/#!/app/andvisio, accessed on 24 May 2023) [37,38]. The ANDSystem incorporates utilities for mining knowledge from PubMed published scientific texts, and it has been chosen to search for mTOR signaling pharmacological modulators. Using the ANDVisio tool, we have built three networks based on three fragments of the mTOR pathway: PI3K/Akt (Figure 7), Ras (Figure 8), and p38 MAPK (Figure 9), along with related drug-type objects. Then drug-type objects associated with AD but not with ASD have been identified.

By means of the ANDVisio tool, both pharmacological and natural mTOR regulators with potential therapeutic relevance for ASD have been identified, such as propofol, dexamethasone, celecoxib, statins, berberine, resveratrol, quercetin, myricetin, myo-inositol, and several amino acids.

In many cases, the mechanisms of action of pharmacological mTOR inhibitors are well investigated. Propofol reduces the activity of the mTOR signaling pathway by inhibiting mTOR/eIF4E [39]. Dexamethasone regulates the Ras, PI3K/Akt, and mTOR pathways by activating AKT [40] and inhibiting ERK [41]. Celecoxib regulates the PI3K/Akt mTOR pathways by activating AKT and inhibiting mTOR [42]. Celecoxib has also shown efficacy for ASD treatment when taken with risperidone in a randomized controlled trial [43]. Statins, such as simvastatin and atorvastatin, inhibit PI3K/Akt and mTOR [44].

It has recently been shown that many biologically active additives are natural mTOR regulators. Berberine regulates the p38 MAPK, Ras, and mTOR pathways by activating p38 and ERK [45]. Resveratrol regulates the PI3K/Akt and mTOR pathways by inhibiting AKT [46]. Quercetin regulates the PI3K/Akt and mTOR pathways by inhibiting PI3K [47,48]. Moreover, resveratrol and quercetin have already been tested for ASD treatment [49,50]. Myricetin regulates the MAPK, PI3K/Akt, and mTOR pathways [51]. Myo-inositol inhibits PI3K and enhances the stability of active mTOR [52,53]. Among the amino acids, leucine, isoleucine, and valine activate mTOR, while histidine, lysine, and threonine inhibit it [54].

Thus, the ANDVisio tool of the ANDSystem has proven to be an effective tool for directly identifying substances that modulate a known signaling pathway such as mTOR. In addition, these findings show that an in-depth bioinformatic analysis of diagnostic full-exome sequencing data from ASD patients and the search for signaling pathways linking genes predisposed to autism may lead to the discovery of new targets for mechanism-based therapies.

## 4. Materials and Methods

### 4.1. Extracting Genes from Diverse Data Sources and Gene-Set Analysis

The following sets of genes were selected for analysis:

1. Genes implicated in autism susceptibility (from SFARI Gene database released 20 October 2022 [22])—1095 genes;
2. Autism predisposition genes (41588_2022_1104_MOESM3_ESM.xlsx [20])—185 genes;
3. Autism and developmental delay predisposition genes (ASD/DD) (41588_2022_1104_MOESM3_ESM.xlsx [20])—664 genes;
4. Genes predisposing to Alzheimer’s disease (13195_2017_252_MOESM2_ESM.doc [55])—430 genes;
5. Alzheimer’s disease predisposition genes (from the ADVP database [21])—956 genes;
6. FMRP target genes (1-s2.0-FMRP_tags_842-mmc2.xls [56]—842 genes and Jansen2017.xlsx [57]—1047 genes)—1614 genes;
7. Genes included in the mTOR signaling network (Table S1.xlsx [23]—248 genes and KEGG database [58]—153 genes)—341 genes;
8. mTOR-sensitive genes (mTOR-sensitive 5UTR.xlsx [59])—6543 genes;
9. Genes associated with autoimmune diseases (from the GAAD [60])—4186 genes.
10. All protein-coding genes of the human genome for which PAI and DI values were calculated [24]—19478 genes.

The sets of genes 1, 6, 7, and 8 are exhaustively described in [61]. The gene sets 2 and 3 were obtained as an analysis of full-exome sequencing of the genomes of more than 150,000 individuals diagnosed with neurodevelopmental disorders [20]. The sets of genes 4 and 5, associated with Alzheimer’s disease, were taken from original article [55] and from Alzheimer’s Disease Variant Portal (ADVP) [21]. The gene set 9 was taken from A Gene and Autoimmiune Disease Association Database (GAAD) [60]. The complete lists of all analyzed gene sets are given in Supplementary Table S1.

An online service (https://bioinformatics.psb.ugent.be/webtools/Venn/, accessed on 20 May 2024) was used to find intersections of genes from different sets and build Venn diagrams.

### 4.2. Phylostratigraphic Analysis and Divergence Analysis

Phylostratigraphy is a method used to determine the evolutionary origin of genes by identifying moments in genome evolution marked by a sharp increase in the number of new genes and by detecting genes that are unique to certain taxa [62,63]. This method relies on calculating the oldest taxon in which an ortholog of the studied gene can be identified, thereby indicating the gene’s evolutionary origin.

In our analysis, we have begun by retrieving orthologous genes from the KEGG Orthology database [64]. The taxon reflecting the gene’s age has been determined by identifying the most distantly related lineage in which orthologs are present. For each gene, the Phylostratigraphy Age Index (PAI) [34] has been calculated according to the distance of this taxon from the root of the phylogenetic tree (Table 2). A larger PAI value indicates that the gene diverged more recently (i.e., younger genes), whereas a smaller PAI value corresponds to a more ancient origin.

Evolutionary variability of each gene has been further characterized using the Divergence Index (DI), derived from the ratio of nonsynonymous to synonymous substitutions (dN/dS). For each gene and its orthologs from the Hominidae family, specifically orthologs found in the western lowland gorilla *Gorilla gorilla gorilla*, Sumatran orangutan *Pongo abelii*, and common chimpanzee *Pan troglodytes*. The dN/dS ratio for each ortholog pair has been subsequently normalized to derive the DI, which reflects the type of selection on gene sequence within said taxon. A DI value ranging from 0 to 1 indicates that a gene is undergoing stabilizing selection, a value near 1 indicates neutral evolution, and a value much greater than 1 indicates positive selection.

All analyses of evolutionary indices have been conducted within the Orthoweb software package (v1.0.0) [65]. The calculation of PAI has used the method based on KEGG orthology groups. Resulting data have been exported and processed using custom Python scripts (v3.10.1) for downstream statistical analysis and visualization.

### 4.3. Network Construction

The construction of gene networks has been performed using the STRING database (v11.5), as well as the ANDVisio tool of the ANDSystem.

The STRING database systematically collects and combines data on protein–protein interactions: both physical and functional. The data comes from a number of sources: automated analysis of scientific literature, calculated predictions of interaction based on coexpression, conservative genomic context, databases of interaction experiments, and known complexes/pathways from carefully selected sources [66]. The data is accessed through the website https://string-db.org/.

ANDVisio (https://www-bionet.sscc.ru/andvisio/#!/app/andvisio (accessed on 24 May 2023)) is a software tool that allows searching, visualizing, editing, and saving associative gene networks in various formats that describe the relationships between molecular genetic objects such as proteins, genes, and metabolites, biological processes, and diseases. ANDVisio supports filtering by object types, relationships between objects and information sources, and provides tools for graph layout, the shortest path search, and cycle detection in graphs https://www-bionet.sscc.ru/andvisio/#!/app/about (accessed on 24 May 2023) [37,38].

## Figures and Tables

**Table 1:** Average values of PAI and DI indices for genes involved in autism, neurodevelopmental disorders, Alzheimer’s disease predispositions, and mTOR signaling pathway.

Gene Set Number	Gene Sets	PAI	DI
1	Genes implicated in autism susceptibility (from SFARI Gene database) [22]	2.86	0.24
2	Autism predisposition genes [20]	2.80	0.16
3	Autism and developmental delay predisposition genes (ASD/DD) [20]	2.59	0.15
5	Alzheimer’s disease predisposition genes (from the ADVP database) [21]	3.18	0.33
7	Genes included in the mTOR signaling network [23]	2.29	0.18
10	All protein-coding human genes [24]	3.29	0.38

**Table 2:** PAI values and taxonomic units dating the corresponding phylostratigraphic age of genes.

PAI	Phylostratum
1	Cellular Organisms
2	Eukaryota
3	Metazoa
4	Chordata
5	Craniata
6	Vertebrata
7	Euteleostomi
8	Mammalia
9	Eutheria
10	Euarchontoglires
11	Primates
12	Haplorrhini
13	Catarrhini
14	Hominidae
15	Homo
16	*Homo sapiens*

## Data Availability

Data is contained within the article and Supplementary Materials.

## Supplementary Materials

The supporting information can be downloaded at: https://www.mdpi.com/article/10.3390/ijms262010066/s1.

## Abbreviations

The following abbreviations are used in this manuscript:

ASD	Autism Spectrum Disorder
AD	Alzheimer’s disease
SFARI	Simon’s Foundation Autism Research Initiative
mTOR	mechanistic target of rapamycin
FMRP	fragile X mental retardation protein
PAI	Phylostratigraphic Age Index
DI	Divergence Index
DD	developmental delay

## References

[1] Center for Disease Control and Prevention. https://www.cdc.gov/autism/data-research/?CDC_AAref_Val=https://www.cdc.gov/ncbddd/autism/data.html.

[2] Soysal P., Tan S.G. (2021). The prevalence and co-incidence of geriatric syndromes in older patients with early-stage Alzheimer’s disease and dementia with Lewy bodies. Aging Clin. Exp. Res..

[3] Rajan K.B., Weuve J., Barnes L.L., McAninch E.A., Wilson R.S., Evans D.A. (2021). Population estimate of people with clinical Alzheimer’s disease and mild cognitive impairment in the United States (2020–2060). Alzheimers. Dement..

[4] Mencer S., Kartawy M., Lendenfeld F., Soluh H., Tripathi M.K., Khaliulin I., Amal H. (2021). Proteomics of autism and Alzheimer’s mouse models reveal common alterations in mTOR signaling pathway. Transl. Psychiatry.

[5] Hughes H.K., Mills Ko E., Rose D., Ashwood P. (2018). Immune Dysfunction and Autoimmunity as Pathological Mechanisms in Autism Spectrum Disorders. Front. Cell. Neurosci..

[6] Ashwood P., van deWater J. (2004). Is autism an autoimmune disease?. Autoimmun. Rev..

[7] Edmiston E., Ashwood P., van de Water J. (2017). Autoimmunity, Autoantibodies, and Autism Spectrum Disorder. Biol. Psychiatry.

[8] DiStasio M.M., Nagakura I., Nadler M.J., Anderson M.P. (2019). T lymphocytes and cytotoxic astrocyte blebs correlate across autism brains. Ann. Neurol..

[9] Chen X., Holtzman D.M. (2022). Emerging roles of innate and adaptive immunity in Alzheimer’s disease. Immunity.

[10] Meier-Stephenson F.S., Meier-Stephenson V.C., Carter M.D., Meek A.R., Wang Y., Pan L., Chen Q., Jacobo S., Wu F., Lu E. (2022). Alzheimer’s disease as an autoimmune disorder of innate immunity endogenously modulated by tryptophan metabolites. Alzheimer’s Dement. Transl. Res. Clin. Interv..

[11] Shuid A.N., Jayusman P.A., Shuid N., Ismail J., Kamal N.N., Naina Mohamed I. (2020). Update on Atypicalities of Central Nervous System in Autism Spectrum Disorder. Brain Sci..

[12] Delgoffe G.M., Pollizzi K.N., Waickman A.T., Heikamp E., Meyers D.J., Horton M.R., Xiao B., Worley P.F., Powell J.D. (2011). The kinase mTOR regulates the differentiation of helper T cells through the selective activation of signaling by mTORC1 and mTORC2. Nat. Immunol..

[13] Liu Y., Zhang D.T., Liu X.G. (2015). mTOR signaling in T cell immunity and autoimmunity. Int. Rev. Immunol..

[14] Ortiz-González X.R. (2021). Mitochondrial Dysfunction: A Common Denominator in Neurodevelopmental Disorders?. Dev. Neurosci..

[15] Lenzi P., Ferese R., Biagioni F., Fulceri F., Busceti C.L., Falleni A., Gambardella S., Frati A., Fornai F. (2021). Rapamycin Ameliorates Defects in Mitochondrial Fission and Mitophagy in Glioblastoma Cells. Int. J. Mol. Sci..

[16] Thellung S., Corsaro A., Nizzari M., Barbieri F., Florio T. (2019). Autophagy Activator Drugs: A New Opportunity in Neuroprotection from Misfolded Protein Toxicity. Int. J. Mol. Sci..

[17] Richardson A., Galvan V., Lin A.L., Oddo S. (2015). How longevity research can lead to therapies for Alzheimer’s disease: The rapamycin story. Exp. Gerontol..

[18] Caccamo A., Majumder S., Richardson A., Strong R., Oddo S. (2010). Molecular interplay between mammalian target of rapamycin (mTOR), amyloid-beta, and Tau: Effects on cognitive impairments. J. Biol. Chem..

[19] Narayanan U., Nalavadi V., Nakamoto M., Thomas G., Ceman S., Bassell G.J., Warren S.T. (2008). S6K1 phosphorylates and regulates fragile X mental retardation protein (FMRP) with the neuronal protein synthesis-dependent mammalian target of rapamycin (mTOR) signaling cascade. J. Biol. Chem..

[20] Fu J.M., Satterstrom F.K., Peng M., Brand H., Collins R.L., Dong S., Wamsley B., Klei L., Wang L., Hao S.P. (2022). Rare coding variation provides insight into the genetic architecture and phenotypic context of autism. Nat. Genet..

[21] Kuksa P.P., Liu C.L., Fu W., Qu L., Zhao Y., Katanic Z., Clark K., Kuzma A.B., Ho P.C., Tzeng K.T. (2022). Alzheimer’s Disease Variant Portal: A Catalog of Genetic Findings for Alzheimer’s Disease. J. Alzheimers. Dis..

[22] Abrahams B.S., Arking D.E., Campbell D.B., Meord H.C., Morrow E.M., Weiss L.A., Menashe I., Wadkins T., Banerjee-Basu S., Packer A. (2013). SFARI Gene 2.0: A community-driven knowledgebase for the autism spectrum disorders (ASDs). Mol. Autism.

[23] Caron E., Ghosh S., Matsuoka Y., Ashton-Beaucage D., Therrien M., Lemieux S., Perreault C., Roux P.P., Kitano H. (2010). A comprehensive map of the mTOR signaling network. Mol. Syst. Biol..

[24] Piovesan A., Antonaros F., Vitale L., Strippoli P., Pelleri M.C., Caracausi M. (2019). Human protein-coding genes and gene feature statistics in 2019. BMC Res. Notes.

[25] Feng L., Wang G., Song Q., Feng X., Su J., Ji G., Li M. (2024). Proteomics revealed an association between ribosome-associated proteins and amyloid beta deposition in Alzheimer’s disease. Metab. Brain Dis..

[26] Liu P.P., Han X., Li X., Dai S.K., Xu Y.J., Jiao L.F., Du H.Z., Zhao L.H., Li R.F., Teng Z.Q. (2025). An EED/PRC2-H19 Loop Regulates Cerebellar Development. Adv. Sci..

[27] Liu J., Yuan S., Niu X., Kelleher R., Sheridan H. (2022). ESR1 dysfunction triggers neuroinflammation as a critical upstream causative factor of the Alzheimer’s disease process. Aging.

[28] Barral S., Reitz C., Small S.A., Mayeux R. (2014). Genetic variants in a ‘cAMP element binding protein’ (CREB)-dependent histone acetylation pathway influence memory performance in cognitively healthy elderly individuals. Neurobiol. Aging.

[29] Zhu Y., Huang Y., Tang T., Xie Y. (2024). HDAC1 and HDAC2 orchestrate Wnt signaling to regulate neural progenitor transition during brain development. iScience.

[30] Lewerissa E.I., Nadif Kasri N., Linda K. (2024). Epigenetic regulation of autophagy-related genes: Implications for neurodevelopmental disorders. Autophagy.

[31] Xie P.-L., Zheng M.-Y., Han R., Chen W.-X., Mao J.-H. (2024). Pharmacological mTOR inhibitors in ameliorating Alzheimer’s disease: Current review and perspectives. Front. Pharmacol..

[32] Ehninger D., Han S., Shilyansky C., Zhou Y., Li W., Kwiatkowski D.J., Ramesh V., Silva A.J. (2008). Reversalof learning deficits in a Tsc2+/− mouse model of tuberoussclerosis. Nat. Med..

[33] Pérez-Cano L., Azidane Chenlo S., Sabido-Vera R., Sirci F., Durham L., Guney E. (2023). Translating precision medicine for autism spectrum disorder: A pressing need. Drug Discov. Today.

[34] Mustafin Z.S., Lashin S.A., Matushkin Y.G. (2021). Phylostratigraphic analysis of gene networks of human diseases. Vavilovskii Zhurnal Genet. Selektsii.

[35] Nagy P.L., Griesenbeck J., Kornberg R.D., Cleary M.L. (2002). A trithorax-group complex purified from Saccharomyces cerevisiae is required for methylation of histone H3. Proc. Natl. Acad. Sci. USA.

[36] Li S., Qiao Y., Di Q., Le X., Zhang L., Zhang X., Zhang C., Cheng J., Zong S., Koide S.S. (2009). Interaction of SH3P13 and DYDC1 protein: A germ cell component that regulates acrosome biogenesis during spermiogenesis. Eur. J. Cell Biol..

[37] Demenkov P.S., Ivanisenko T.V., Kolchanov N.A., Ivanisenko V.A. (2011). ANDVisio: A new tool for graphic visualization and analysis of literature mined associative gene networks in the ANDSystem. Silico Biol..

[38] Ivanisenko V.A., Saik O.V., Ivanisenko N.V., Tiys E.S., Ivanisenko T.V., Demenkov P.S., Kolchanov N.A. (2015). ANDSystem: An Associative Network Discovery System for automated literature mining in the field of biology. BMC Syst. Biol..

[39] Wang Z., Cao B., Ji P., Yao F. (2021). Propofol inhibits tumor angiogenesis through targeting VEGF/VEGFR and mTOR/eIF4E signaling. Biochem. Biophys. Res. Commun..

[40] Bossmann M., Ackermann B.W., Thome U.H., Laube M. (2017). Signaling Cascade Involved in Rapid Stimulation of Cystic Fibrosis Transmembrane Conductance Regulator (CFTR) by Dexamethasone. Int. J. Mol. Sci..

[41] Morita M., Suyama H., Igishi T., Shigeoka Y., Kodani M., Hashimoto K., Takeda K., Sumikawa T., Shimizu E. (2007). Dexamethasone inhibits paclitaxel-induced cytotoxic activity through retinoblastoma protein dephosphorylation in non-small cell lung cancer cells. Int. J. Oncol..

[42] Zhang P., He D., Song E., Jiang M., Song Y. (2019). Celecoxib enhances the sensitivity of non-small-cell lung cancer cells to radiation-induced apoptosis through downregulation of the Akt/mTOR signaling pathway and COX-2 expression. PLoS ONE.

[43] Asadabadi M., Mohammadi M.R., Ghanizadeh A., Modabbernia A., Ashrafi M., Hassanzadeh E., Forghani S., Akhondzadeh S. (2013). Celecoxib as adjunctive treatment to risperidone in children with autistic disorder: A randomized, double-blind, placebo-controlled trial. Psychopharmacology.

[44] Lashgari N.A., Roudsari N.M., Zadeh S.S.T., Momtaz S., Abbasifard M., Reiner Ž., Abdolghaffari A.H., Sahebkar A. (2023). Statins block mammalian target of rapamycin pathway: A possible novel therapeutic strategy for inflammatory, malignant and neurodegenerative diseases. Inflammopharmacology.

[45] Okubo S., Uto T., Goto A., Tanaka H., Nishioku T., Yamada K., Shoyama Y. (2017). Berberine Induces Apoptotic Cell Death via Activation of Caspase-3 and -8 in HL-60 Human Leukemia Cells: Nuclear Localization and Structure-Activity Relationships. Am. J. Chin. Med..

[46] Sun X., Zhang Y., Wang J., Wei L., Li H., Hanley G., Zhao M., Li Y., Yin D. (2010). Beta-arrestin 2 modulates resveratrol-induced apoptosis and regulation of Akt/GSK3ß pathways. Biochim. Biophys. Acta.

[47] Russo M., Milito A., Spagnuolo C., Carbone V., Rosén A., Minasi P., Lauria F., Russo G.L. (2017). CK2 and PI3K are direct molecular targets of quercetin in chronic lymphocytic leukaemia. Oncotarget.

[48] Lim J.Y., Lee J.Y., Byun B.J., Kim S.H. (2015). Fisetin targets phosphatidylinositol-3-kinase and induces apoptosis of human B lymphoma Raji cells. Toxicol. Rep..

[49] Marchezan J., Deckmann I., da Fonseca G.C., Margis R., Riesgo R., Gottfried C. (2022). Resveratrol Treatment of Autism Spectrum Disorder-A Pilot Study. Clin. Neuropharmacol..

[50] Alvarez-Arellano L., Salazar-García M., Corona J.C. (2020). Neuroprotective Effects of Quercetin in Pediatric Neurological Diseases. Molecules.

[51] Song X., Tan L., Wang M., Ren C., Guo C., Yang B., Ren Y., Cao Z., Li Y., Pei J. (2021). Myricetin: A review of the most recent research. Biomed. Pharmacother..

[52] Xiong D., Pan J., Zhang Q., Szabo E., Miller M.S., Lubet R.A., You M., Wang Y. (2017). Bronchial airway gene expression signatures in mouse lung squamous cell carcinoma and their modulation by cancer chemopreventive agents. Oncotarget.

[53] Rameh L.E., York J.D., Blind R.D. (2025). Inositol phosphates dynamically enhance stability, solubility, and catalytic activity of mTOR. J. Biol. Chem..

[54] van Sadelhoff J.H.J., Perez Pardo P., Wu J., Garssen J., van Bergenhenegouwen J., Hogenkamp A., Hartog A., Kraneveld A.D. (2019). The Gut-Immune-Brain Axis in Autism Spectrum Disorders; A Focus on Amino Acids. Front. Endocrinol..

[55] Hu Y.S., Xin J., Hu Y., Zhang L., Wang J. (2017). Analyzing the genes related to Alzheimer’s disease via a network and pathway-based approach. Alzheimers Res. Ther..

[56] Darnell J.C., Van Driesche S.J., Zhang C., Hung K.Y., Mele A., Fraser C.E., Stone E.F., Chen C., Fak J.J., Chi S.W. (2011). FMRP stalls ribosomal translocation on mRNAs linked to synaptic function and autism. Cell.

[57] Jansen A., Dieleman G.C., Smit A.B., Verhage M., Verhulst F.C., Polderman T.J.C., Posthuma D. (2017). Gene-set analysis shows association between FMRP targets and autism spectrum disorder. Eur. J. Hum. Genet..

[58] Kanehisa M., Goto S. (2000). KEGG: Kyoto encyclopedia of genes and genomes. Nucleic Acids Res..

[59] Gandin V., Masvidal L., Hulea L., Gravel S.P., Cargnello M., McLaughlan S., Cai Y., Balanathan P., Morita M., Rajakumar A. (2016). nanoCAGE reveals 5′ UTR features that define specific modes of translation of functionally related MTOR-sensitive mRNAs. Genome Res..

[60] Lu G., Hao X., Chen W.H., Mu S. (2018). GAAD: A Gene and Autoimmiune Disease Association Database. Genom. Proteom. Bioinform..

[61] Trifonova E.A., Klimenko A.I., Mustafin Z.S., Lashin S.A., Kochetov A.V. (2019). The mTOR Signaling Pathway Activity and Vitamin D Availability Control the Expression of Most Autism Predisposition Genes. Int. J. Mol. Sci..

[62] Domazet-Loso T., Brajković J., Tautz D. (2007). A phylostratigraphy approach to uncover the genomic history of major adaptations in metazoan lineages. Trends Genet..

[63] Domazet-Lošo M., Široki T., Šimičević K., Domazet-Lošo T. (2024). Macroevolutionary dynamics of gene family gain and loss along multicellular eukaryotic lineages. Nat. Commun..

[64] Kanehisa M., Furumichi M., Tanabe M., Sato Y., Morishima K. (2017). KEGG: New perspectives on genomes, pathways, diseases and drugs. Nucleic Acids Res..

[65] Ivanov R.A., Mukhin A.M., Kazantsev F.V., Mustafin Z.S., Afonnikov D.A., Matushkin Y.G., Lashin S.A. (2025). Orthoweb: A Software Package for Evolutionary Analysis of Gene Networks. Vavilovskii Zhurnal Genet. Selektsii.

[66] Szklarczyk D., Gable A.L., Lyon D., Junge A., Wyder S., Huerta-Cepas J., Simonovic M., Doncheva N.T., Morris J.H., Bork P. (2019). STRING v11: Protein-protein association networks with increased coverage, supporting functional discovery in genome-wide experimental datasets. Nucleic Acids Res..

