# Supplementary figures and images for: Genetic and Pathogenic Overlaps Between Autism Spectrum Disorder and Alzheimer’s Disease: Evolutionary Features and Opportunities for Drug Repurposing
Source: Int J Mol Sci. 2025 Oct 16;26(20):10066. doi: 10.3390/ijms262010066 (PMC12564320; doi:10.3390/ijms262010066)

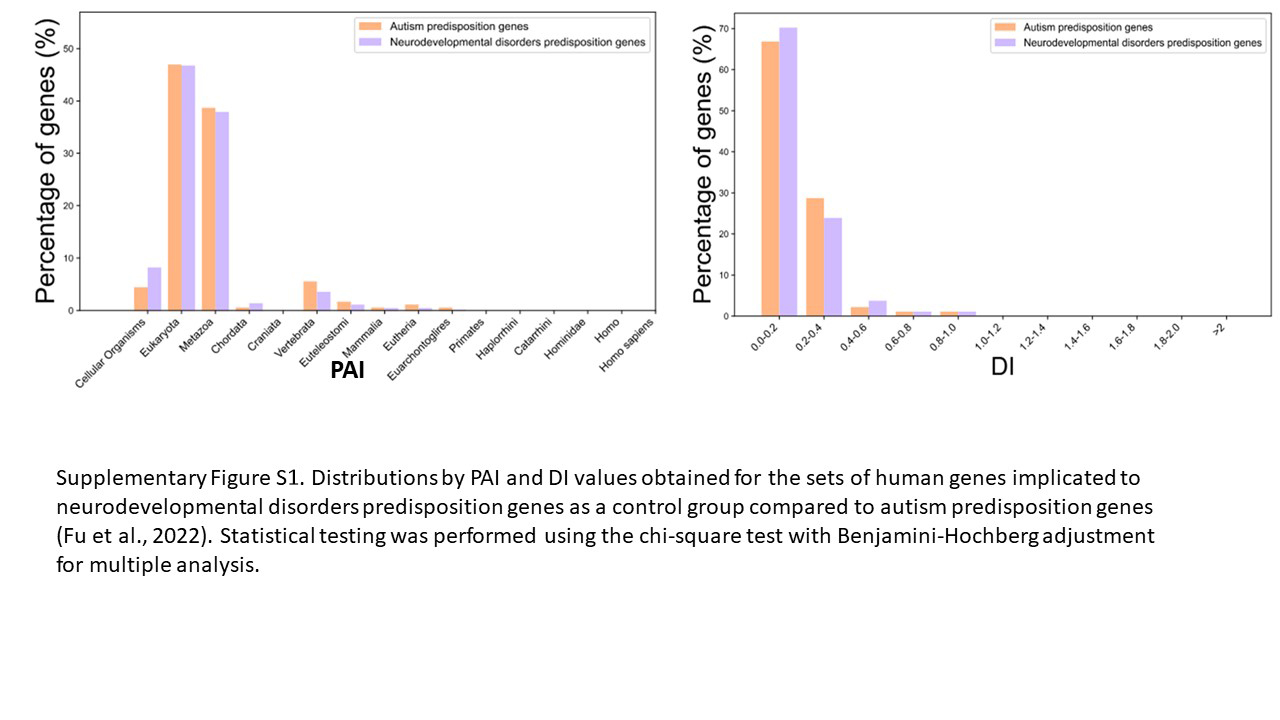

Supplement: Supplementary file 1 [file ijms-26-10066-s001.zip › Supplementary Figure S1.jpg]
